# Reduced resting-state connectivity in areas involved in processing of face-related social cues in female adolescents with atypical anorexia nervosa

**DOI:** 10.1038/s41398-018-0333-1

**Published:** 2018-12-13

**Authors:** Gaia Olivo, Ingemar Swenne, Christina Zhukovsky, Anna-Kaisa Tuunainen, Helena Salonen-Ros, Elna-Marie Larsson, Santino Gaudio, Samantha J. Brooks, Helgi B. Schiöth

**Affiliations:** 1 Department of Neuroscience, Functional Pharmacology, Uppsala University, Uppsala, Sweden; 2 Department of Women’s and Children’s Health, Uppsala University, Uppsala, Sweden; 3 Department of Neuroscience, Child and Adolescent Psychiatry, Uppsala University, Uppsala, Sweden; 4 Department of Surgical Sciences, Radiology, Uppsala University, Uppsala, Sweden; 5 Centre for Integrated Research (CIR), Area of Diagnostic Imaging, Università “Campus Bio-Medico di Roma”, Rome, Italy; 6 Department of Human Biology, University of Cape Town, Cape Town, South Africa

## Abstract

Atypical anorexia nervosa (AN) has a high incidence in adolescents and can result in significant morbidity and mortality. Neuroimaging could improve our knowledge regarding the pathogenesis of eating disorders (EDs), however research on adolescents with EDs is limited. To date no neuroimaging studies have been conducted to investigate brain functional connectivity in atypical AN. We investigated resting-state functional connectivity using 3 T MRI in 22 drug-naïve adolescent patients with atypical AN, and 24 healthy controls. Psychological traits related to the ED and depressive symptoms have been assessed using the Eating Disorders Examination Questionnaire (EDE-Q) and the Montgomery–Åsberg Depression Rating Scale self-reported (MADRS-S) respectively. Reduced connectivity was found in patients in brain areas involved in face-processing and social cognition, such as the left putamen, the left occipital fusiform gyrus, and specific cerebellar lobules. The connectivity was, on the other hand, increased in patients compared with controls from the right inferior temporal gyrus to the superior parietal lobule and superior lateral occipital cortex. These areas are involved in multimodal stimuli integration, social rejection and anxiety. Patients scored higher on the EDE-Q and MADRS-S questionnaires, and the MADRS-S correlated with connectivity from the right inferior temporal gyrus to the superior parietal lobule in patients. Our findings point toward a role for an altered development of socio-emotional skills in the pathogenesis of atypical AN. Nonetheless, longitudinal studies will be needed to assess whether these connectivity alterations might be a neural marker of the pathology.

## Introduction

The Diagnostic and Statistical Manual of Mental Disorders, fifth edition (DSM-5) defined atypical anorexia nervosa (AN) as the presence of all of the criteria for AN, except for a less severe weight loss. Despite patients with atypical AN are in the near normal-weight range at presentation^1,2^, atypical AN can result in significant morbidity and mortality^1^. Atypical AN has a high incidence in adolescents^3^, and can represent up to 33% of the diagnoses of eating disorders (EDs) in adolescent medicine clinics^1^. The medical consequences are comparable to those reported for full-syndrome AN^2^, and patients can present signs of starvation and medical instability^1^. Given the similarities between AN and atypical AN, a debate has arisen as to whether atypical AN is an entity to be separated from AN^4^.

Neuroimaging could improve our knowledge regarding the pathogenesis of EDs, leading to a better understanding of the complex interrelation between neurobiological and psychosocial aspects of these disorders^5^. This might help designing effective treatment strategies, targeting specific dysfunctions and neurobehavioral features underlying specific EDs^5^. This is particularly important in adolescents, given the plasticity their brain undergoes during development. Functional connectivity in fact fluctuates during adolescence^6^, with patterns typically reflecting a middle stage between childhood and adulthood^6^. In this context, increases and decreases in functional connectivity reflect the strengthening of some networks, paralleled by the weakening of other connections^6^. Research on adolescents with EDs is however limited. The few studies available have been conducted in full-syndrome AN patients aged 16-25, reporting the involvement of the reward network^7–10^. Specifically, altered connectivity has been found in the thalamo-frontal^8^ and accumbo-frontal circuitry^7^. The increase in accumbo-frontal connectivity, in particular, persisted after weight restoration^7^. The local connectivity of the thalamus and insula^9,10^ has also been found to be altered. Alterations of the resting-state connectivity in the cortico-limbic circuitry and the insula have also been reported in the adults with AN^11^, particularly in areas involved in cognitive control and visual and homeostatic integration^11^. Moreover, the only study conducted on a sample of solely adolescents ( < 18 years) reported reduced connectivity in the executive control network in patients with full-syndrome AN compared with controls^12^.

To date, no neuroimaging studies, in either adults or adolescents, have been conducted in atypical AN to investigate brain circuits that potentially underlie the development of ED. We have thus investigated resting-state functional connectivity and ED-related and depressive symptoms in 22 drug-naïve adolescent patients with atypical AN, and 24 healthy controls. We hypothesized that patients would show functional connectivity alterations similar to those observed in full-syndrome AN, given the similarities between the two disorders in terms of pathogenesis, genetics, and clinical features^2,4^.

## Methods

### Subjects

All participants and their guardians gave written consent to participate in the study. All procedures contributing to this work comply with the ethical standards of the relevant national and institutional committees on human experimentation and with the Helsinki Declaration of 1975, as revised in 2008. The protocol was approved by the regional ethics committee (Etikprövningsnämnderna) of Uppsala, Sweden.

Twenty-four adolescent female outpatients (mean age 14.5 years, range 12.5–17.3 years) were recruited by the Eating Disorder Unit (EDU) of the Department of Child and Adolescent Psychiatry at the Uppsala University Hospital, Uppsala, Sweden. They were diagnosed with atypical AN, according to the fifth edition of the DSM-5^13^, as they presented with features of AN but were above -2 body mass index (BMI) standard deviations (SDS) for age^14^. BMI percentile per age was also calculated, according to the World Health Organization reports (https://www.who.int/growthref/bmifa_girls_5_19years_per.pdf?ua=1) (Table 1). The initial assessment of patients and the diagnostic procedure were performed by a pediatrician with experience of ED and associated to an ED clinic, and followed a structured protocol including: the history of the ED, medical history (including medical and psychiatric comorbidities), menstrual status, demographics, and physical examination (including weight and height measurements). Diagnoses of atypical AN were confirmed by a psychiatrist at the EDU. All patients were also assessed with the diagnostic instruments included in the “Stepwise” data collection system^15^. The “Stepwise” data collection system was introduced in Sweden since 2014 and is used by all specialized ED services. It comprises semi-structured diagnostic interviews, clinical ratings and self-ratings, automated follow-up schedules and administrative functions^15^. Patients were also administered the MINI-KID interview^16^ to further screen for comorbid diseases. Furthermore, the history of weight and height changes was obtained from the growth charts provided by the school health services. Patients started treatment after the diagnosis was made. The treatment consisted of a family-based intervention, aiming at helping the parents to take a leading role against the ED. Parents were given advice on regular meals and meal sizes, however a standardized diet for all patients was not provided, as the family-based intervention requires to be tailored to the specific family necessities and situation. Twenty-nine healthy controls (mean age 14.8 years, range 13.0–18.0 years) were recruited from local schools through advertisement.

**Table 1:** Clinical and demographics data of the participants

	Patients *mean (SD)*	Controls *mean (SD)*	*p* value
Age (years)	14.5 (0.34)	14.8 (0.29)	0.503
BMI at scan (Kg/m^2^)	19.50 (2.54)	19.9 (0.34)	0.198
BMI percentile per age (at scan)	42.61 (25.44)	46.83 (21.70)	0.460
EDE-Q	2.7 (0.31)	0.2 (0.04)	0.001**
MADRS-S	25.4 (2.68)	5.6 (1.21)	0.001**
Disease Duration (years)	0.6 (0.39)	—	—
BMI at diagnosis (Kg/m^2^)	18.6 (0.52)	—	—
BMI % per age (at diagnosis)	33.77 (26.91)	—	—
BMI-SDS at diagnosis	-0.27 (1.13)	—	—

^*^p < 0.05; **p < 0.01

ED-related cognition was assessed via a 38-items self-reported questionnaire, the EDE-Q^17^, youth version^18^. The EDE-Q comprises four subscales measuring specific features of the ED behavior: Restraint, Eating Concern, Shape Concern, Weight Concern. Depression symptoms were assessed with the MADRS-S^19^.

Exclusion criteria for all participants were male gender, comorbid neurological diseases, left-handedness, metallic implants, claustrophobia and use of psychotropic medication, past or current comorbidity (patients) or history (controls) of psychiatric disorders. Approximately 50% of new patients were excluded according to these criteria. For controls, additional exclusion criteria were a BMI < −2 BMI-SDS and an EDE-Q total score > 2.0, which has been suggested as the optimal cut-off to distinguish between the clinical and the general population^20^.

### MRI acquisition

The scanning procedure was carried out within 40 days of the initial visit at the clinic. The diagnosis of atypical AN was still confirmed by the psychiatrist at the time of scanning. A Philips 3-Tesla scanner (Achieva, Philips Healthcare, Best Netherlands) using a standard 32-channel head coil was used to acquire the MRI sequences. Structural images were acquired with a T1-weighted turbo-field-echo (TFE) sequence (TR = 8100 ms; TE = 3.7 ms; flip angle: 8°; slice thickness = 1 mm; slice spacing = 1 mm). 180 resting-state volumes were registered during the T2*-weighed echo-planar imaging (EPI) sequence (TR = 2000 ms; TE = 30 ms; flip angle: 90°; slice thickness = 3 mm; slice spacing = 3.9 mm; slices number = 32).

### Pre-processing of imaging data

Pre-processing was carried out with Data Processing Assistant for Resting-state fMRI Advanced (DPARSFA; http://rfmri.org/) extension in Statistical Parametric Mapping 12 (SPM12; http://www.fil.ion.ucl.ac.uk/spm/software/spm12/ Wellcome Trust Centre for Neuroimaging, University College London) implemented in MATLAB (version r2017a). The first ten volumes were discarded to allow for signal equilibration. Slice timing was performed and the functional T2 images were realigned to correct for head motion. A cut-off of 3 mm was used to exclude participants due to excessive motion. Two patients and five controls were excluded; twenty-two patients and twenty-four controls were retained for further analyses.

The structural T1 images were co-registered to the functional images, and the DARTEL (Diffeomorphic Anatomical Registration Through Exponentiated Lie Algebra)^21^ option was chosen to segment the structural images in grey matter (GM), white matter (WM) and cerebrospinal fluid (CSF) maps. A band-pass filtering was applied to the functional scans (0.01–0.1 Hz) to remove residual motion and physiological artefactual effects from the BOLD signal. The functional images were then normalized to the standard anatomical Montreal Neurological Institute (MNI) template^22^ using 2 × 2 × 2 mm voxel size. Images were smoothed with a 4 full width at half maximum (FWHM) Gaussian kernel to increase the signal to noise ratio and to accommodate for anatomical and functional variability between subjects.

Functional and structural images were imported in the functional connectivity toolbox CONN (https://www.nitrc.org/projects/conn). Data were denoised by regressing out the effect of WM, CSF and motion parameters. Finally, linear detrending was performed. First level statistical analyses were performed with CONN. Motion parameters were entered as first level covariates. CONN provides a complete brain parcellation including 91 cortical areas and 15 subcortical areas from the FSL Harvard-Oxford Atlas, and 26 cerebellar areas from the AAL atlas, for a total of 132 seeds. Functional connectivity maps were imported in Statistical Parametric Mapping toolbox (SPM 12) (http://www.fil.ion.ucl.ac.uk/spm/) for second level analyses.

### Statistical analysis of clinical and demographic data

Statistical analyses of clinical and demographic data were performed with Statistical Package for Social Science (SPSS), v. 24 (https://www.ibm.com/analytics/data-science/predictive-analytics/spss-statistical-software). Data were checked for normality of the distribution with the Shapiro Wilk’s test. A *t* test for independent samples was carried out to test for differences between patients and controls on age and BMI percentile per age. No differences between group variances were found at the Levene’s test for equality of variance. EDE-Q total score and MADRS-S score were found to be not normally distributed, thus a Mann-Whitney test was applied. The threshold for significance was set at *p* < 0.05.

### Statistical analysis of imaging data

Imaging data were analyzed using SPM 12 (http://www.fil.ion.ucl.ac.uk/spm/). A one-way ANCOVA was performed to test for differences in connectivity between patients and controls. Age and BMI were entered as covariates of no interest. Each of the 132 seeds was tested separately. The primary voxel-level threshold was set at *p* < 0.001^23^. A further correction for Family-wise error rate (FWE) at cluster level was applied to voxels surviving the primary threshold. Correction for multiple testing according to Bonferroni’s approach was also applied to the cluster-level threshold to account for the number of seeds. The final threshold was then set at *p* < 0.0004 (0.05/132), FWE-corrected. The post-hoc *t* tests were masked for the main effect.

The connectivity correlation coefficients of the areas found to be significant at the between-groups comparison were extracted and imported in SPSS for correlation analyses. Separate multiple regression analyses were run for the EDE-Q and MADRS-S total scores, leading to 8 tests (2 measures × 4 clusters). The analyses were performed separately in patients and controls. The threshold for significance was thus set at *p* < 0.006, to account for multiple testing according to Bonferroni. All analyses were corrected for age and BMI at the time of scanning.

## Results

### Clinical and demographic data

Age, BMI and BMI percentile per age at the time of scanning were not significantly different between patients and controls. Patients and controls differed significantly on the EDE-Q (0.001) and MADRS-S (*p* < 0.001) scores (Table 1).

### Functional connectivity differences between groups

**Fig. 1:**
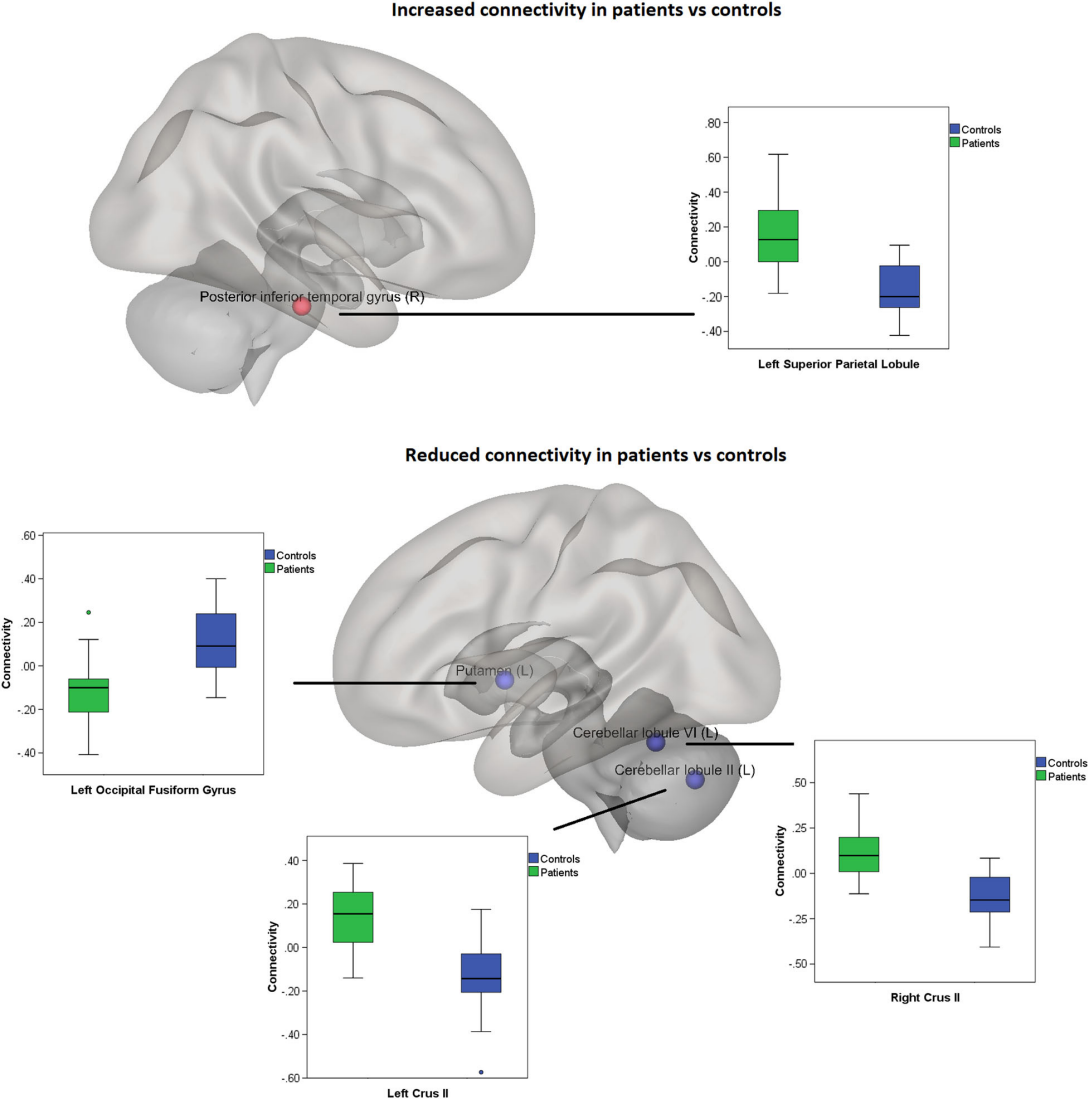
**Connectivity differences between patients and controls**. The figure represents connections where increased (upper panel) or decreased (lower panel) connectivity was found in patients compared with controls. The seeds were overlayed on a brain surface rendering with CONN. The bar graphs represent the group differences in connectivity toward the specific clusters. 95% confidence intervals are reported in each graph

Patients showed reduced functional connectivity from the left cerebellar VI lobule to the right vermis (*p* < 0.00003, FWE-corrected); from the left cerebellar II lobule to the left crus II (*p* < 0.0003); and from the left putamen to the left occipital fusiform gyrus (*p* < 0.0002, FWE-corrected), extending to the lingual gyrus. The connectivity was, on the other hand, increased in patients compared with controls from the right posterior inferior temporal gyrus (ITG) to the left superior parietal lobule (SPL) extending to the lateral superior occipital cortex (sLOC) (Fig. 1, Table 2).

**Table 2:** Functional connectivity differences in patients compared with controls

Seed	Size	*x*	*y*	*z*	*P* value	Structure	Sign	*T*	*p* value
L, Cerebellum VI	175	2	−76	−30	0.00003	R Vermis Crus II	−	5.84	0.0002
L, Putamen	135	−22	−68	−10	0.0002	L Occipital Fusiform	−	4.60	0.001
L, Cerebellum II	138	−2	−76	−38	0.0003	L Vermis Crus II	−	6.06	0.001
R, pITG	143	−30	−66	58	0.0002	L SPL	+	5.75	0.001

“−” Connectivity reduced in patients compared with controls,  "+" connectivity increased in patients compared with controls

*L* left, *R* right, *pITG* posterior inferior temporal gyrus, *SPL* superior parietal lobule

### Correlations between clinical measures and functional connectivity

**Fig. 2:**
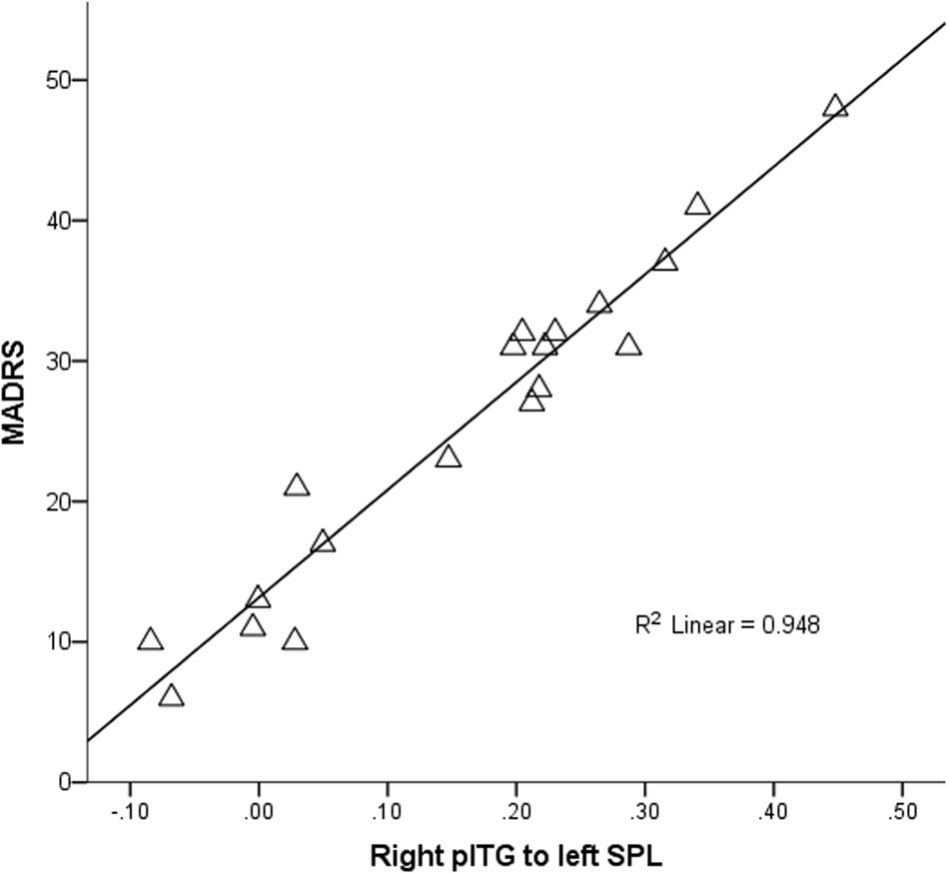
**Correlations between MADRS-S and connectivity**. The figure shows the correlations between the MADRS-S total score and the connectivity from the right posterior inferior temporal gyrus (pITG) to the left superior parietal lobule (SPL). The predicted values from the regression models were extracted and plotted agains the MADRS-S with SPSS

Within patients, a significant positive correlation was found between the connectivity from the right pITG to the left SPL and the MADRS-S score (*p* < 0.005) (Fig. 2). No correlations were found in controls.

## Discussion

We explored functional connectivity and clinical ED-related and depressive symptoms in a sample of 22 drug-naïve adolescents recently diagnosed with atypical AN, scanned within 40 days of diagnosis, and 24 healthy controls. Patients showed reduced cerebellar functional connectivity as well as reduced connectivity from the left putamen to the left occipital fusiform gyrus. The connectivity was, on the other hand, increased in patients compared with controls from the right pITG to the left SPL and SLOC. Moreover, patients scored higher on the EDE-Q and MADRS-S questionnaires.

The anterior cerebellum, encompassing the lobules II-V and part of the lobule VI, is defined as the sensorimotor cerebellum and is connected to the sensorimotor cortical areas^24,25^. In particular, the representation of hands and orofacial movements occurs in these lobules^26^. In our sample, patients exhibited a reduced connectivity from the lobules VI and II to crus II in the vermis, involved in higher-order cognitive processing^25^. The cerebellar lobule VI is involved in executive function and emotional processing^24,25^, while the lobule II has somatosensory functions^27^ and stores a representation of the homunculus^28^. Lobule VI in particular has been involved in the development of social interactions and in environmental learning^29^. Patients also showed reduced connectivity between the left putamen and the left occipital fusiform gyrus, extending to the lingual gyrus. Putamen and fusiform gyrus connectivity have been reported to be reduced in acute AN patients^10^, and both areas are hyperactive in response to negative facial stimuli and hypoactive in response to happy facial stimuli in depressed patients^30^. Indeed, the fusiform gyrus is quite specific to the visual processing of facial expressions^30,31^, and activity in the putamen is elicited by happy human faces, having social rewarding value in the context of a normal development^32^. Overall, the reduction in functional connectivity in our atypical AN patients affected mostly areas involved in face processing and social cognition, suggesting a core role for an altered development of socio-emotional skills.

The connectivity was, on the other hand, increased in patients compared with controls from the right pITG to the left SPL and SLOC. The ITG and SLOC are part of the ventral visual stream^33^, responsible for object recognition and discrimination^29,34,35^. The occipitotemporal visual stream is highly interconnected with the striatum, and these cortico-striatal loops are involved in visual discrimination learning based on the reinforcement versus extinction of stimulus-response associations (habit formation)^33^. Both the pITG and the SPL are involved in the higher-order integration between different features of the same visual stimulus, as well as in the integration between different sensory modalities^36,37^. Interestingly, the ITG has been involved in aesthetic judgements^38^ and in processing faces’ features^39,40^ and identities^41,42^, and an increased volume of the ITG has been related with sensitivity to social rejection^43^. Moreover, increased resting-state connectivity of the right ITG, correlating with anxiety scores, has been found in patients with somatization disorder^44^. Depressed patients also showed increased fractional amplitude of low-frequency fluctuation during resting-state, reflective of the intensity of spontaneous brain activity, in the right ITG^45^. This is also in line with the correlation we found between the MADRS-S score and the connectivity between these structures in patients. Importantly, the SPL is part of the mirror neuron system and is involved in self-processing and in the theory of the mind^46^. Thus, the increased connectivity between these areas in patients might reflect a dysfunctional emotional and social development, characterized by increased anxiety toward social rejection and enhanced tendency toward judging others compared to own self.

Patients also scored higher on the EDE-Q and MADRS-S questionnaires. Whether the neural alterations precede and cause the onset of the ED-related or depressive symptoms, or are a consequence of the same, remains to be assessed. However, it is worth noticing that none of our patients was diagnosed with depression at the time of enrollment in the study, as comorbidities or history of other psychiatric disorders rather than the ED was set as an exclusion criterion. Future longitudinal studies will nonetheless be necessary to investigate this issue in depth.

Of note, the alterations we found in atypical AN are not overlapping with the scarce previous literature on AN. The only study investigating anorectic adolescents, in fact, reported decreased functional connectivity solely in the executive control network^12^. On the other hand, few other studies including young adults with AN (16-25 years) have reported altered connectivity in the reward network, specifically in the accumbo-frontal^7^, thalamocortical^8^ and thalamo-insular networks^9,10^. The fronto-parietal network has also been involved^47^. Our study was however the first investigation of the neural correlates underlying atypical AN in adolescents. We focused on a homogeneous sample including only drug-naïve and comorbidities-free females with a restrictive subtype of atypical AN. Future studies will thus be needed to compare atypical AN and full-threshold AN.

Our study has some limitations. The sample size was small though adequate for fMRI studies^48^, calling for future studies to verify our findings. We did not have information pertaining the menstrual status of the controls, thus we could not control our findings for menstrual status. Finally, the controls did not undergo a complete psychological examination, and their mental health history was self-reported. However, using the subclinical cut-off on the EDE-Q as exclusion criterion ensured the exclusion of controls with possible underdiagnosed EDs. It also has to be noticed that the scanning procedure was performed 10 to 40 days after diagnosis. This delay was necessary due to the informed consent protocol. In fact, at their first visit to the clinic, parents and their children were given information about the study and they had one week time to communicate whether they wanted to participate or not. Then, a time for the MRI was set, in accordance to the hospital necessities. The treatment was however started right after the diagnosis due to ethical reasons.

## Conclusion

We investigate functional brain connectivity and ED-related and depressive symptoms, as measured by the EDE-Q and MADRS-S questionnaires respectively, in a sample of 22 drug-naive female adolescents diagnosed with atypical AN, and 24 healthy controls. We report reduced connectivity in patients in brain areas involved in face-processing and social cognition, while an increased connectivity, correlating with depressive symptoms, was found in areas involved in multimodal integration of sensory stimuli, aesthetic judgment, and social rejection anxiety. Our findings point toward a core role for an altered development of socio-emotional skills in the pathogenesis of atypical AN. Nonetheless, longitudinal studies will be needed to assess whether these functional alterations might be a neural marker of the pathology.
